# Health literacy and its effect on chronic disease prevention: evidence from China’s data

**DOI:** 10.1186/s12889-020-08804-4

**Published:** 2020-05-14

**Authors:** Lefan Liu, Xujun Qian, Zhuo Chen, Tianfeng He

**Affiliations:** 1 Center for Health Economics, School of Economics, University of Nottingham Ningbo China, Ningbo, 315100 China; 2 Department of Health and Management, Ningbo First Hospital, Ningbo, 315010 China; 3 College of Public Health, University of Georgia, Athens, GA 30606 USA; 4 Department of Health Education, Ningbo Municipal Center for Disease Control and Prevention, 237 Yongfeng Street, Ningbo, 315010 China

**Keywords:** Health literacy, Chronic disease prevention, Risk perception, Comorbidity, China, Rural-urban disparity

## Abstract

**Background:**

Improving health literacy is an important public health goal in many countries. Although many studies have suggested that low health literacy has adverse effects on an individual’s health outcomes, confounding factors are often not accounted. This paper examines the interplay between health literacy and chronic disease prevention.

**Methods:**

A population-based sample of 8194 participants aged 15–69 years old in Ningbo were used from China’s 2017 National Health Literacy Surveillance Data. We use multivariate regression analysis to disentangle the relationship between health literacy and chronic disease prevention.

**Results:**

We find the association between health literacy and the occurrence of the first chronic condition is attenuated after we adjust the results for age and education. This might arise because having one or more chronic conditions is associated with better knowledge about chronic diseases, thus improve their health literacy. More importantly, we find health literacy is associated with a reduction in the likelihood of having a comorbid condition. However, this protective effect is only found among urban residents, suggesting health literacy might be a key factor explaining the rural-urban disparity in health outcomes.

**Conclusion:**

Our findings highlight the important role of health literacy in preventing comorbidities instead of preventing the first chronic condition. Moreover, family support could help improve health literacy and result in beneficial effects on health.

## Background

Health literacy is a topic with growing importance in the field of public health. It has been defined in many different ways since it was first introduced as a term in 1974 [1]. Here we use a widely accepted definition that was developed by the (US) National Library of Medicine and used by (US) Health People 2010 (the document that reports the government’s national health objectives): “the degree to which individuals can obtain, process, and understand the basic health information and services they need to make appropriate health-related decisions” [1]. Low health literacy is often a significant health challenge in many countries. For example, in 2003 National Assessment of Adult Literacy data (the only national data on health literacy skills in US), it is reported that over one third of adults in US had limited health literacy [2]. In Europe, a 2013 WHO report shows nearly half of all Europeans have inadequate or problematic health literacy [3].^1^ Although a significant proportion of the general population have low health literacy, rates of limited health literacy are often higher among some socioeconomically disadvantaged groups, e.g. the elderly, individuals who have not completed high school, and people living in poverty [2, 4, 5].

A growing body of literature looks at the relationship between health literacy and health outcomes. Although the strength of evidence remains insufficient, many of these studies find that lower health literacy is associated with poorer outcomes, including low health knowledge, increased incidence of chronic illness, poorer intermediate disease markers, and insufficient use of preventive health services [6–9]. As a result, promoting health literacy is now a public health goal in many countries and interventions to improve health literacy are often prioritized [10, 11].

This is particularly true for China. In 2008, the Chinese government conducted a survey titled “National Health Literacy Surveillance” (NHLS) [11]. The first nationwide surveillance on adult health literacy, the NHLS surveyed around 80,000 residents aged 15–69 and included 96 items intended to measure health literacy. The survey was based on a government statement published in earlier 2008 titled “Basic Knowledge and Skills of People’s Health Literacy (pilot edition)” [12], which is also called “66 Tips of Health: Chinese Resident Health Literacy Manual”. The first NHLS data showed that the national health literacy rate in 2008 is merely 6.48%. This rate measures the percentage of residents who were able to give correct answers to at least 80% of the survey questions, i.e., having adequate health literacy. A large rural-urban disparity exists with the rate of health literacy being 9.49% among urban residents and only 3.43% among rural residents [13]. The second national survey was conducted in 2012 and the survey has been conducted every year since then. This rate of health literacy rose steadily from 8.8% in 2012 to 10.25% in 2015. In 2016, the Chinese government issued its “Healthy China 2030 Blueprint”, which proposed major health indicators to be achieved in 2030, such as average life expectancy, infant mortality rate, and mortality rate of children below 5 years old. In this blueprint, the rate of national health literacy is aimed to increase to 30%, tripling the existing level in 2015.

However, policymakers often face a challenge in that knowledge does not necessarily induce actual behavioural change, which is medicated by many factors [2]. Indeed, one of the key factors could be education as better educated people can better internalise health information [14]. Besides, attitudes, social norms and self-efficacy are also responsible for most of behaviour intention that leads to subsequent behaviour change [15, 16]. The differences in these intermediate outcomes might result in different effects that health literacy plays in improving health outcomes.

Another strand of literature studies the extent to which a negative health event can prompt individuals to adopt risk-reducing behaviours [17]. One of the studies, for example, explored whether having cancer or having a family member with cancer was associated with intention to quit smoking [18]. They found having a family member with cancer was associated with a smoker’s intention to quit due to an elevated level of perceived cancer risk. Similarly, the diagnosis of first chronic disease might expose individuals to more disease-related information and increase their risk perceptions in developing a new chronic condition. Thus, the relationship between health literacy and chronic disease prevention can be affected by whether or when the respondent has had their first chronic disease. This has not been explicitly studied in the literature and is what we aim to study in this paper. We start by examining the correlation between health literacy and chronic disease occurrence. More importantly, we explore the role that health literacy has on preventing comorbid chronic conditions.

## Methods

### Data and sample selection

Our data comes from a sub-sample of the 2017 NHLS conducted in Ningbo, which is one of the developed cities in the eastern coastal regions of China. It is representative of the permanent residents aged 15–69 years old who lived in Ningbo for more than 6 of the previous 12 months at the time of the survey regardless of whether they have local household registration (*hukou*). Residents living in military bases, hospitals, prisons, nursing homes, and dormitories are excluded.

We used a stratified multi-stage PPS (probabilities proportional to population size) sampling frame. The sampling strategy follows the national guideline [19] and a description using similar strategies can be found in an earlier study [20]. At each 12 counties (or county-level cities) in Ningbo, we selected 4 streets (or townships), and then selected 2 neighbourhood-committees (or villages) within each street (or township).^2^ If there were greater than 750 but less than 1500 households within a neighbourhood-committee (or village), the unit was regarded as a primary sampling unit (PSU). If the selected neighbourhood-committee (or village) had more than 1500 households, it was divided into several units, each containing roughly 750 households, and one of the units was randomly selected and used as a PSU. In each PSU, our mappers constructed a list of households by field trips, from which 120 households were randomly selected. One permanent resident aged 15–69 was then chosen randomly in each household.^3^ In each PSU, at least 83 respondents were interviewed and a total of 8299 respondents were surveyed. All respondents who agreed to participate in the survey signed an informed consent form at the beginning of the survey. The sampling weights were calculated based on the five-stage sampling process. In what follows our working sample includes 8149 respondents (aged 15–69) surveyed in 2017, of whom we have complete information on the variables of interest.

### Questionnaire design and measure of health literacy

The questionnaire was developed based on “Basic Knowledge and Skills of People’s Health Literacy (pilot edition)”, which was designed by experts in public health, health education and promotion, and clinical medicine using the Delphi method [22]. The final questionnaire was compiled by the National Institute of Health Education of National Health Commission in China. The questionnaire kept the same format since 2012 and included similar instruments used in the second NHLS. A study [20] using the 2012 NHLS data of 3731 participant in Hunan (a province in South China) assessed the reliability, construct validity, and measurement invariance of the national health literacy scale. The overall Cronbach’s alpha of the original scale (80 items) was 0.95 and the Spearman-Brown split-half coefficient 0.94 [20].^4^ In addition, the questionnaire was completed by face-to-face interviews with the selected respondent in each household. Double data entry is used to maintain strict quality control.

The health literacy questions cover three dimensions: (1) knowledge and attitudes (22 items); (2) behaviour and lifestyle (16 items); and (3) health-related skills (12 items). There are four types of questions: true-or-false; single-answer (only one correct answer in multiple-choice questions); multiple-answer (more than one correct answer in multiple-choice questions); and vignette questions. Vignette questions were given following a paragraph of instruction or medical information. Correct response to each true-or-false and single-answer questions counts one and correct response to a multiple-answer question counts two (a correct response had to contain all the correct answers and no wrong ones) towards the total score and the full score is 66.

To compute the rate of health literacy, a respondent is defined as having adequate health literacy if their total score is at least 80% of the full score (i.e. 53). This method has been used consistently over time in China and we follow this convention for ease of interpretation but will use raw scores for robustness tests. It is worth noting the questionnaires used in 2008 and 2012 surveys are different, and pre-tests were conducted to make sure the resulting rates are comparable.^5^ Questions, depending on their relevance to public health, can be divided into six categories: (1) scientific views of health; (2) infectious disease prevention; (3) chronic disease prevention; (4) safety and first aid; (5) medical care; and (6) health information.

Threshold for each category is pre-defined (80% of the full score in each category) to classify the health literacy level of an individual in each category. Here, we use the sub-scale “health literacy on chronic disease prevention (CDP)” to examine the relationship between health literacy and chronic disease occurrence. The score of an individual’s health literacy on CDP is obtained from answering 9 questions involving both “the knowledge and attitude” (e.g. understanding that vegetables cannot be replaced by fruits; and adolescents can also have depression) and “behaviour and lifestyle” (e.g. understanding of self-monitored blood pressure; and early warning signs of cancer) dimensions of health literacy. We present the original 9 items in Table S1 in the Additional file 1 together with the rate of correction for each question among 8194 respondents in our sample. If a respondent gave correct responses to all 9 questions, their score would be 12. An individual is classified as having adequate health literacy on CDP if their score obtained is 10 or above (80% of the full score). We will use the raw score (ranges from 0 to 12) for robustness check.

Our survey also collected basic information of the respondents on their demographic characteristics and health condition. In particular, respondents were asked whether they had any chronic disease and the type of the disease if any, including hypertension, heart problems, cerebrovascular diseases, diabetes, malignant tumour (cancer) and other. Respondents were also asked the number of years since their first chronic disease was diagnosed. These questions asked in the survey are presented in Table S2 in the Additional file 1.

### Statistical analyses

We use the binary outcome whether a respondent has any chronic disease as dependent variable and examine the effect of health literacy in a multivariate regression model and control for the demographic and socio-economic status factors of the respondent, including region of residence, gender, annual income, number of household members, occupation, age and level of education. To ease interpretation, a linear probability model (LPM) is used to estimate the model. In robustness section, however, we will show our results are mainly the same using a nonlinear model such as logit. We use Stata 15.0 for statistical analyses.

## Results

### Characteristics of the respondents

Table 1 presents summary statistics for our sample disaggregated by region of residence (rural/urban). We report four statistics: mean, standard deviation, minimum and maximum value for each variable. In the final column, we report the *p*-value to test the equality of the means between the rural and urban samples.

**Table 1:** Summary statistics

Sample	All				Rural	Urban	Rural vs Urban
Number of Observations	*N* = 8194				*n* = 4192	*n* = 4002	
Variables	mean	s.d.	min/max		mean	mean	*p*-value
**Key variables of interest**							
Any chronic diseases (=1)	0.261	0.439	0	1	0.293	0.227	0.000
Hypertension (=1)	0.189	0.392	0	1	0.215	0.162	0.000
Heart problems (=1)	0.019	0.138	0	1	0.021	0.018	0.322
Cerebrovascular disease (=1)	0.007	0.082	0	1	0.008	0.006	0.243
Diabetes (=1)	0.049	0.215	0	1	0.055	0.042	0.007
Cancer (=1)	0.007	0.086	0	1	0.009	0.006	0.218
Other chronic diseases (=1)	0.036	0.186	0	1	0.040	0.032	0.074
Adequate health literacy on CDP^a^ (=1)	0.258	0.438	0	1	0.231	0.286	0.000
**Demographics**							
Region (=1 urban)	0.488	0.500	0	1	0.000	1.000	0.000
Has local hukou (=1)	0.928	0.259	0	1	0.965	0.890	0.000
Gender (=1 male)	0.489	0.500	0	1	0.493	0.484	0.424
Age in years	48.925	13.242	15	69	51.372	46.363	0.000
1:Aged 15–44	0.342	0.474	0	1	0.245	0.443	0.000
2:Aged 45–59	0.389	0.488	0	1	0.430	0.347	0.000
3:Aged 60–69	0.269	0.443	0	1	0.325	0.210	0.000
Household size	2.825	1.520	1	50	2.877	2.771	0.002
Annual income (1000 CNY)	86.552	103.176	0	3500	71.462	102.359	0.000
**Education level**							
1:Illiterate	0.086	0.281	0	1	0.132	0.038	0.000
2:Elementary	0.274	0.446	0	1	0.364	0.179	0.000
3:Middle school	0.300	0.458	0	1	0.309	0.291	0.067
4:High school	0.168	0.374	0	1	0.117	0.222	0.000
5:College or above	0.172	0.377	0	1	0.078	0.270	0.000
**Occupation**							
1:Working in public sectors	0.093	0.290	0	1	0.061	0.127	0.000
2:Farmers	0.289	0.453	0	1	0.449	0.121	0.000
3:Manual labourers	0.184	0.388	0	1	0.191	0.177	0.117
4:Working in private sectors	0.172	0.378	0	1	0.094	0.254	0.000
5:Other	0.262	0.440	0	1	0.206	0.321	0.000

Note: The *p*-value is calculated using either the t-test (if continuous) or the proportion test (if binary); a pre-test of equality of variance is also conducted. ^a^ CDP refers to chronic disease prevention

Firstly, we look at the demographic characteristics. Our sample is evenly split between rural and urban areas (48% are urban residents) and about half are men. A typical respondent is aged 49, who lives in a household of 3 members and self-reported an annual income of 86,552 CNY ($ 12,364).^6^ In terms of education, about 9% of the respondents are illiterate, 17% finished high school, and 17% have a college degree or above. In terms of occupation, about 9% are working in public sectors (including civil servants, medical workers, and teachers), 29% are farmers, 18% are manual labourers, and 17% are working in private sectors. It should be noted that our respondents are likely to have a distribution of the socio-economic background that is better than the national average because Zhejiang province, where Ningbo belongs to, has a real GDP per capita above the national average.

We now turn to their health literacy and conditions of chronic diseases. Table 1 shows that the rate of health literacy on CDP is 25.8%, meaning out of 100 people living in Ningbo, 26 are able to give correct answers to 80% or more of questions presented in Table S1 in the Additional file 1 and be considered as having adequate health literacy on CDP. This figure is much higher than the reported 2017 national level (15.7%) but similar as that for Shanghai (24.2%) [24].^7^ The prevalence rate for chronic disease is 26%.^8^ The most prevalent disease type is hypertension (19%) followed by diabetes (5%) and heart problems (2%). The prevalence rate for cerebrovascular diseases or cancer is not high, about 1%.^9^

Significant differences also arise in rural and urban samples in terms of health literacy and chronic diseases. The urban residents have a higher level of health literacy on CDP. At the same time, they have fewer chronic diseases. Urban residents are also significantly younger (46 vs 51 years), which we think is partly due to the rural-urban migration, where younger people from rural areas move to urban areas for better job opportunities. Urban residents tend to live with fewer household members (2.9 vs 2.8). They earn more annually (102359 vs 71462 CNY or $14622 vs $10208) and are better educated (19% vs 49% in terms of the proportion of high school or above). Not surprisingly, they are also more likely to work in public sectors and are less likely to work as farmers.

### Characteristics of groups with different level of health literacy

From Table 1, we find urban residents are significantly better-off: they are healthier and have a higher level of health literacy on CDP; and they are younger, better educated and wealthier. In order to investigate the relationship between health literacy and chronic disease occurrence, we further group our respondents by their level of health literacy on CDP in Table 2 to examine their respective characteristics.

**Table 2:** Summary statistics by level of health literacy on CDP

Sample	Health literacy		*p*-value
	Adequate	Inadequate	
Number of observations	*n* = 2114	*n* = 6080	
Variables	mean	mean	
**Key variables of interest**			
Any chronic diseases (=1)	0.225	0.273	0.000
Hypertension (=1)	0.164	0.198	0.001
Heart problems (=1)	0.021	0.019	0.437
Cerebrovascular disease (=1)	0.005	0.007	0.291
Diabetes (=1)	0.039	0.052	0.018
Cancer (=1)	0.007	0.008	0.610
Other chronic diseases (=1)	0.031	0.038	0.132
Adequate health literacy on CDP^a^ (=1)	1.000	0.000	/
**Demographics**			
Region (=1 urban)	0.541	0.470	0.000
Has local hukou (=1)	0.939	0.924	0.023
Gender (=1 male)	0.484	0.490	0.659
Age in years	45.236	50.208	0.000
1:Aged 15–44	0.457	0.301	0.000
2:Aged 45–59	0.360	0.400	0.002
3:Aged 60–69	0.182	0.299	0.000
Household size	2.934	2.787	0.000
Annual income (1000 CNY)	106.421	79.643	0.000
**Education level**			
1:Illiterate	0.053	0.098	0.000
2:Elementary	0.177	0.308	0.000
3:Middle school	0.257	0.315	0.000
4:High school	0.206	0.155	0.000
5:College or above	0.307	0.125	0.000
**Occupation**			
1:Working in public sectors	0.153	0.072	0.000
2:Farmers	0.244	0.304	0.000
3:Manual labourers	0.172	0.188	0.090
4:Working in private sectors	0.220	0.156	0.000
5:Other	0.211	0.280	0.000

Note: The p-value is calculated using either the t-test (if continuous) or the proportion test (if binary); a pre-test of equality of variance is also conducted. ^a^ CDP refers to chronic disease prevention

Not surprisingly, we find the prevalence of chronic diseases is significantly lower among the group with adequate health literacy. In addition, this more ‘literate’ group are more likely to live in the urban areas, are younger (45 vs 50), have a higher income, are better educated, and more likely to work in public sectors or employed in private sectors.^10^ Similar patterns are observed in the rural and the urban samples (See Table S4 in the Additional file 1).

While we observe a lower prevalence rate of chronic diseases among residents with adequate health literacy, we also find they are younger, better educated, and wealthier, which are all factors that are associated with a lower likelihood of having chronic diseases. In other words, the negative relationship we observe between rate of health literacy and chronic disease prevalence may not reflect the causal effect that health literacy has on chronic disease prevention, but actually reflect the observed characteristics, such as age and education have on having chronic diseases.^11^ Next, we will take into account these ‘confounders’ to untangle the relationship between health literacy and chronic diseases.

### The effect of health literacy on chronic diseases occurrence

We predict the occurrence of chronic disease with a set of hierarchical equations in Table 3. In column (1) we include no covariate but the binary variable of health literacy alone. In columns (2)–(4), we add sequentially three blocks of variables to the equations, representing, in order of entry, region of residence, gender, income and household size; occupation; age and education. This ordering provides a means to observe how each block of variables added later could explain the effect of health literacy shown in column (1). In column (5) we included the full set of covariates rendering us a ‘purer’ effect of health literacy, which partials out potential confounders.

**Table 3:** OLS estimates on having any chronic disease

Dep: Has any chronic disease	(1)	(2)	(3)	(4)	(5)
Sample	All	All	All	All	All
Adequate health literacy on CDP (=1)	−0.048***	− 0.034***	−0.025**	0.018*	0.018*
	(0.011)	(0.011)	(0.011)	(0.011)	(0.011)
Region (=1 urban)		−0.059***			0.018*
		(0.010)			(0.010)
Gender (=1 male)		0.002			−0.000
		(0.010)			(0.009)
Annual income (log)		−0.024***			−0.010***
		(0.003)			(0.003)
Household size		−0.027***			−0.005*
		(0.003)			(0.003)
*Occupation (Base: 1:Public sectors)*					
2:Farmers			0.244***		0.020
			(0.018)		(0.020)
3:Manual labourers			0.108***		0.023
			(0.019)		(0.020)
4:Private sectors			0.034*		0.011
			(0.019)		(0.018)
5:Other			0.120***		−0.009
			(0.018)		(0.019)
*Age group (Base: 1:Aged 15–44)*					
2:Aged 45–59				0.200***	0.198***
				(0.012)	(0.012)
3:Aged 60–69				0.403***	0.397***
				(0.015)	(0.015)
*Education (Base: 1:Illiterate)*					
2:Elementary				− 0.025	− 0.024
				(0.018)	(0.018)
3:Middle				−0.065***	−0.060***
				(0.018)	(0.019)
4:High school				−0.063***	−0.056***
				(0.020)	(0.022)
5:College or above				−0.081***	−0.071***
				(0.022)	(0.024)
Observations	8194	8194	8194	8194	8194
R-squared	0.002	0.025	0.038	0.153	0.155

Note: The dependent variable is a binary variable indicating whether a respondent has any chronic disease (=1 if has any chronic disease, 0 otherwise). Estimates on the constant are not reported. ***p < 0.01, ** *p* < 0.05, * *p* < 0.1. Standard errors in parentheses

The first equation in column (1) reveals that adequate health literacy on CDP is associated with a reduction in the likelihood of having chronic disease by 4.8 percentage points. This merely replays what we observed in Table 2. The second equation in column (2), which added gender, annual income and number of household members, shows that higher income is also associated with a lower likelihood of having chronic diseases and the effect of health literacy remains negative despite a small reduction in magnitude. The effect of household size is also significant, showing that respondents living in a larger household are less likely to report having chronic diseases. Results in column (3) show that occupation is also a strong predictor of the respondent’s chronic condition. Compared to those working in public sectors, farmers have a higher probability of having chronic disease by 24 percentage points, and for manual labourers, this effect is 11 percentage points. More importantly, with the inclusion of occupation, the effect of health literacy is now half the size as before, implying occupation explains away part of the negative effect health literacy has on chronic diseases. In column (4), we include age and education. The effect of health literacy changes sign and is significant at 10% significance level, implying a higher level of health literacy ‘increases’ rather than ‘decreases’ the likelihood of having chronic diseases. The size of this effect is not negligible, about 1.8 percentage points.

The effects of age and education are expected. Those who are younger and better educated are less likely to have chronic diseases. Those effects are significant both statistically and economically, suggesting they are important predictors of having chronic diseases. Also, there is a substantial increase in R-squared in column (4) at the bottom of the table compared to columns (1)–(3), implying age and education are the main confounders to the relationship between health literacy and chronic diseases we observe in column (1). In column (5) we include the full set of covariates and the estimate of health literacy is unaltered compared to column (4).^12^ Similar patterns of results are observed in the split rural and urban samples (Tables S5 and S6 in the Additional file 1).^13^

To further explore how our results vary with the age of the respondents, we split our sample by the age of the respondent and the results are reported in Table S7. We find the positive association between health literacy and chronic disease occurrence is only present among those aged 60–69 but is absent in the two younger age groups.^14^

### The effect of chronic disease on health literacy

In this section we explicitly estimate a model that predicts the probability that a respondent has adequate ‘health literacy on CDP’. Again, we carry out this task using LPM and our main results are reported in Table 4. Differing results arise in rural and urban samples and we discuss first the urban results as a benchmark in Panel A and then highlight differences in rural results in Panel B in Table 4.

**Table 4:** OLS estimates on having adequate health literacy on CDP: Disease effects

Dep: Has adequate health literacy on CDP	(1)	(2)	(3)	(4)
**Panel A: Urban sample**				
Any chronic diseases (=1)	0.031*			
	(0.018)			
Duration first chronic disease: One year		0.064**		
		(0.029)		
Duration first chronic disease: 2–4 years		0.026		
		(0.026)		
Duration first chronic disease: 5+ years		0.010		
		(0.027)		
One disease			−0.028	
			(0.019)	
Two+ diseases			0.016	
			(0.039)	
Hypertension (=1)				0.042**
				(0.021)
Observations	4002	3994	4002	4002
R-squared	0.077	0.078	0.077	0.078
**Panel B: Rural Sample**				
Any chronic diseases (=1)	0.010			
	(0.015)			
Duration first chronic disease: One year		0.090***		
		(0.021)		
Duration first chronic disease: 2–4 years		−0.019		
		(0.022)		
Duration first chronic disease: 5+ years		−0.082***		
		(0.026)		
One disease			−0.004	
			(0.016)	
Two+ diseases			0.039	
			(0.032)	
Heart problems (=1)				0.117**
				(0.045)
Observations	4192	4185	4192	4192
R-squared	0.045	0.053	0.046	0.047

Note: Dependent variable is a binary variable indicating the level of health literacy (=1 if has adequate health literacy on CDP, 0 otherwise). Other covariates include gender, annual income, household members, occupation, age and education (and constant). Full list of disease types in column (4) include hypertension, heart problems, cerebrovascular disease, diabetes and cancer and other diseases. Sample size differs in column (2) due to incomplete information provided by respondents on the elapsed time since the first chronic disease was diagnosed. ***p < 0.01, **p < 0.05, *p < 0.1. Standard errors in parentheses

Controlling a series of characteristics of the respondents (gender, annual income, household size, occupation, age and education), we find those with at least one type of chronic disease are significantly more likely to be classified as having adequate health literacy by 3 percentage points (column 1). Given our data is cross-sectional, we cannot say the diagnosis of chronic diseases helps a respondent to access health literacy on CDP unless we could measure the change of health literacy before and after the diagnosis of chronic diseases. Although we do not have such retrospective data, we could compare the level of health literacy between those whose first chronic disease was diagnosed less than 1 year ago and those whose first chronic disease was diagnosed much earlier. This is what we did in our second equation reported in column (2). It shows that among the group whose first chronic disease was diagnosed within the previous year, they are more likely to have adequate health literacy compared to those without chronic diseases. This effect, however, is absent among those whose first chronic disease was diagnosed 2–4 years ago or earlier. Besides, it appears having more chronic conditions increases the likelihood of having adequate health literacy as shown in column (3), but this difference is not statistically significant.^15^

Next, we examine whether this relationship is related to specific type of disease(s). This is done by replacing the number of chronic conditions with six dummy variables indicating the types of diseases in column (4). We find having hypertension is associated with an increase in the likelihood of having adequate health literacy by 4 percentage points (that is 14% increase over 28.6 percentage points - the base rate of health literacy in urban areas). Insignificant results with other disease types are not reported. It is worth noting the effects of diseases with low prevalence such as cancer (less than 1%) may not have been able to be determined in this sample. For these variables, there is insufficient variation, thus a large standard error might arise and less likely can we find a significant result.

Next, we move on to the results for rural sample in Panel B. The results for our rural sample differ significantly from the urban results in terms of the effects of duration and the types of diseases as shown in columns (2) and (4). For rural respondents, those whose first chronic disease was diagnosed more than 5 years ago are significantly less likely to have adequate health literacy on CDP than those without any chronic diseases in column (2). Having heart problems among rural residents is the only disease type that is significantly associated with having adequate health literacy on CDP in column (4).

The effects of other variables have expected signs, which are reported in Table S8 in the Additional file 1. For example, those who work in public sectors are more likely to have adequate health literacy than farmers; older respondents are less likely to have adequate health literacy (but it is only significant in rural areas) and higher education is associated with an increase in the likelihood of having adequate health literacy on CDP. In particular, for the urban sample, we find a positive association between household size and having adequate health literacy on CDP.

### The interaction between health literacy and chronic diseases

Now we are back to the question we asked at the beginning, but in a slightly different form. If being diagnosed with a chronic disease also improves people’s health literacy on CDP, could this improvement reduce the risk of having a new chronic disease? That is to say, does a higher level of health literacy reduce a patient’s likelihood in developing a comorbidity? For example, we might be interested in knowing whether having adequate health literacy reduces the likelihood of having another disease such as hypertension if the patient was diagnosed with diabetes. We will address this question by including the interaction term between health literacy and diabetes and estimate the effect it has on the occurrence of having hypertension. If the interaction is negative, it implies that the effect that health literacy has on hypertension occurrence changes with whether the respondent has had diabetes.^16^

We experimented the above specification alternating the predicting disease variable and the explanatory disease pairs (there are ten of them given we have five types of chronic diseases of interest). We do it for rural sample and urban sample, respectively. We find among urban samples, there are five pairs of disease types that produce a non-negligible interaction effect but not for the rural sample and we report it in Table 5. Separate results for rural sample are available upon request.

**Table 5:** OLS estimates on having comorbid chronic diseases - Urban sample

Dep: Has a specific chronic disease	Cerebro.	Cerebro.	Heart	Diabetes	Diabetes
	(1)	(2)	(3)	(4)	(5)
Sample	Urban	Urban	Urban	Urban	Urban
Adequate health literacy on CDP (=1)	0.001	0.001	0.005	−0.001	−0.001
	(0.003)	(0.003)	(0.005)	(0.007)	(0.007)
Heart problems (=1)	0.064***			0.034	
	(0.011)			(0.028)	
Adequate health literacy on CDP × Heart problems	−0.072***			−0.045	
	(0.021)			(0.055)	
Cancer		0.049***			
		(0.018)			
Adequate health literacy on CDP × Cancer		− 0.059*			
		(0.032)			
Cerebrovascular disease (=1)			0.187***		0.092*
			(0.031)		(0.047)
Adequate health literacy on CDP × Cerebrovascular disease			−0.234***		−0.164
			(0.066)		(0.100)
Observations	4002	4002	4002	4002	4002
R-squared	0.018	0.011	0.032	0.037	0.037

Note: The dependent variable is a binary variable indicating whether the respondent has a specific chronic disease (e.g. =1 if has cerebrovascular disease, 0 otherwise in in column 1). Other covariates in each column include gender, annual income, household members, occupation, age and education (and constant). ***p < 0.01, **p < 0.05, *p < 0.1. Standard errors in parentheses

In columns (1)–(2), we predict the probability of having comorbid cerebrovascular diseases. Expectedly, having heart problems raises the likelihood of cerebrovascular disease by 6 percentage points when an individual does not have adequate health literacy on CDP. The coefficient on health literacy is not significantly different from zero, meaning health literacy has little role to play in preventing an individual from having cerebrovascular diseases as the first chronic disease. However, if an individual has had heart problems, having health literacy reduces the likelihood of having cerebrovascular disease by 7 percentage points. This interaction effect could more than offset the comorbid effect of having heart problems. In column (2), we replace health problems with cancer and again predict the probability of having comorbid cerebrovascular diseases. Having cancer is associated with a higher probability of having cerebrovascular diseases (by 5 percentage points) and the interaction effect is 6 percentage at borderline significance, which again could more than compensate the positive comorbid disease effect.^17^

In columns (3), we predict the probability of having comorbid heart problems with cerebrovascular disease (the reversed case as in column 1). Having cerebrovascular disease is strongly associated with a respondent’s likelihood of having heart problems when the respondent has no health literacy on CDP. The size of interaction effect is considerably large. If a respondent has had cerebrovascular disease, health literacy on CDP is associated with a reduction in the risk of having heart problems by 23.4 percentage points.

In columns (4)–(5), we predict the probability of having comorbid diabetes. The interaction effect is insignificant but sizable, showing health literacy reduces the likelihood of having diabetes by 4 percentage points if a respondent has heart problems. Similarly, health literacy reduces the likelihood of having diabetes by 16.4 percentage points if a respondent has cerebrovascular diseases.

### Sensitivity analyses

In this section, we look into the sensitivity of our main results. We added regional fixed effects (112 dummies indicating neighbourhood-communities/villages) and re-estimated results in Table 5. The results are not altered with the inclusion of regional fixed effects (see Panel A in Table S9 in the Additional file 1). Similar to what we have in Table 5: the interaction effects become greater in size but the significance is not altered, showing our findings are not confounded by the heterogeneity of respondents coming from different neighbourhood-committees/villages.^18^ Next, we apply the sample weights (see Panel B in Table A9 in the Additional file 1). A noticeable difference is the interaction for cancer reduces in size and significance but all else are similar.

In Section 3.5, we analysed the effect of health literacy on several chronic diseases outcomes thus there is a risk of false positives arising from testing multiple hypotheses. If we treated the ten pairs of chronic diseases as independent of each other and with true interaction effect of zero, for α =0.1, the likelihood of finding at least one false positive would be 0.6513.^19^ The likelihood that, as in this paper, five out of ten pairs showed up significant by chance would be mere 0.00149.^20^ However, the chronic diseases outcomes should not be considered uncorrelated. We thus tested our results in a seemingly uncorrelated regression (SUR) framework which allows for correlation between tested outcomes. First proposed by [26], the SUR model is used to estimate a system of linear equations with errors that are correlated across equations for a given individual but are uncorrelated across individuals. We find our results are almost identical to what we reported in Table 5. These results are not reported but available upon request.

Although LPM is easier to interpret, they might suffer from problems such as the error terms will not be normally distributed, there will be heteroskedasticity, and predicted values will fall outside the logical boundaries of 0 and 1. We re-estimated Tables 3 and 4 using logit model and find similar results (reported in Table S10 and Table S11).^21^

Although defining health literacy as a binary outcome is easier for interpretation and comparable with national statistics, we also explore treating level of health literacy on CDP as continuous with scores ranging 0–12 and repeated what we did in Tables 3, 4 and 5. Our key information has not changed. For example, negative effect of health literacy on chronic disease occurrence changes sign and becomes insignificant after controlling for age and education (see Table S12). Hypertension (in urban sample) and heart problems (in rural sample) are found to be significantly associated with a higher score in health literacy on CDP (see Table S13). In particular, among the ten pairs of chronic diseases, we find significantly negative interaction effects among four pairs suggesting health literacy is negatively associated with having a comorbid condition (see Table S14).

## Discussion

We study the relationship between health literacy and chronic disease occurrence among residents aged 15–69 using a population-based sample from the 2017 NHLS data in China. A sub-scale of the national health literacy scale, called “health literacy on chronic disease prevention” is used to measure the level of health literacy and its effect on chronic disease prevention. On average, 25% of the residents in our sample have at least one chronic disease. Although descriptive statistics show people with adequate health literacy are less likely to have any chronic disease, this is mainly driven by the fact that more ‘health literate’ people are also younger and more educated. Once controlling for these differences, we find people with adequate health literacy are more likely to have chronic diseases. This is in line with findings in an extensive systematic review in 2011 [2], showing that the body of evidence on the relationship between health literacy and chronic disease outcomes found mixed results and was limited due to the fact that the majority of studies do not control for potential confounders.

How can we explain this positive association between having health literacy and chronic diseases occurrence once we have controlled for age and education? One possible explanation is that people are more incentivised to acquire the knowledge about the diseases (thus becomes ‘health literate’) in the wake of diagnosis. Other than books, newspapers or magazines, people can access health knowledge from doctors [14]. Therefore, although the estimate is positive, it does not mean having health literacy is bad, but having chronic disease might help a respondent to access health literacy on CDP. If this is the case, we are likely to find a stronger effect among the elderly, who are more vulnerable to chronic diseases. This is consistent with what we found in Table S7 in the Additional file 1: the positive association between health literacy and chronic disease occurrence is only present among those aged 60–69 but is absent in the two younger age groups.^22^ Thus, we do not think this means causally more ‘health literate’ people are more likely to have chronic diseases. Instead, having chronic disease is likely to contribute to the improvement in health literacy.

We move on and explicitly model the probability of having adequate health literacy by controlling for the same set of covariates. We find having chronic disease is associated with a higher level of health literacy and this effect is more pronounced among those whose first chronic disease was diagnosed within the past year (but absent among those whose first chronic disease was diagnosed two or more years ago) and is likely to increase with the number of chronic conditions. We could not rule out the possibility that our results arise due to unobserved factors. Despite this, several potential hypotheses stem from the juxtaposition of these results. First, diagnosis with chronic diseases helps an individual to improve their health literacy on CDP. Second, the improvement on health literacy via this channel however is more likely to occur when a respondent was diagnosed with the disease not long ago. Thirdly, the response to negative health shock also differ by disease type. Improved health literacy is more likely to occur when the respondent was diagnosed with hypertension for the urban resident. Compared to other chronic diseases, the diagnosis of hypertension is reasonably inexpensive and accurate [27] and the relationship between hypertension and several other diseases, such as cerebrovascular disease, heart diseases has been widely accepted. Thus, the implication is an early discovery might be helpful. But we do not find it significant in rural areas, this might be partly attributable to lack of screening, which is common in rural areas.

Besides, the channel through which health literacy increases is likely to be associated with the support of family members because we find household size is positively associated with health literacy. This positive relationship might arise because in a larger household, an individual is more likely to learn health-related information from family members, especially from young members, who have a stronger incentive to acquire new information probably because they are more likely to be better educated or the payoff period for any information investment is longer for them [28]. However, we do not find this positive effect of household size in rural sample. This might occur because rural residents are typically less educated and younger people are attracted to work in urban areas. Therefore, although a rural resident is more likely to live in a larger household than the urban counterpart, their household members are likely to be older and less literate, resulting little gain from living in a larger household. However, it suggests the potential benefit of health literacy promotion via the supportof household members.

Finally, we take into account the interaction between health literacy and chronic disease occurrence to examine the extent to which health literacy helps to prevent chronic diseases. We find if a respondent has had one condition (e.g. heart problems), health literacy might play a protective role in reducing the risk of having a new disease (e.g. cerebrovascular diseases). Strictly speaking, we could not interpret improvement on health literacy as occurring after the first chronic disease was diagnosed because our data is cross-sectional. Despite this, it extends the current literature on the importance of health literacy intervention among patients with chronic diseases [29, 30]. This effect, however, is only present among urban residents. This might arise because rural residents have limited access of health literacy promotion services or are less interested in seeking disease-related information. We did several tests to check the robustness of our finding and found similar results if we accounted for the geographical clustering effect and the correlation between different disease outcomes in a SUR framework.

To the best of our knowledge, this study is the first one to investigate the relationship between health literacy and comorbid chronic diseases using a population-based sample in Ningbo, China, which might shed light to future work in this direction. At the same time, the findings should be interpreted with caution because of the following limitations. First, our data is not national representative and Ningbo is one of the developed coastal cities in China. We do not think our findings will apply to all regions in China given heterogeneous results arise from our urban and rural samples. Further research in other provinces and regions is necessary to understand the relationship between health literacy and comorbid chronic conditions. Second, our work it is based on cross-sectional data, so the causal inferences should be viewed with caution. There could exist unobserved factors that are associated with both health literacy and chronic diseases that are not included in the model, resulting in a spurious relationship. Third, the health literacy measurement we use in this paper is country specific and the instruments included in the questionnaire have been largely the same in the past few years. The former makes it limited in making cross-country comparisons and the latter implies respondents with higher score of health literacy may not be more knowledgeable, but simply better at taking tests than those who achieved lower scores. Lastly, our health literacy measurement can be limited in measuring health literacy, which is an evolving concept [31]. e-health literacy, defined as the ability to appraise health information from electronic sources and apply the knowledge gained to addressing or solving a health problem [32], has attracted rising interests in many countries, but is not incorporated in the NHLS questions. Given more and more people seek health information online to solve their health problems, there is a rising importance in understanding how people deal with (mis)information on social media. According to the China Internet Network Information Centre (CNNIC), approximately 59.6% of Chinese citizens (829 million) in Dec 2018 were internet users compared to 34.3% (457 million) in Dec 2010; a trend that is likely to continue rising [33]. There is an urgent need to include e-health literacy in the future NHLS to better understand the health literacy level among Chinese residents.

Despite these limitations, this study has a number of implications. First, it points to the importance of improving health literacy among people with chronic diseases. Early shock could be a trigger and health workers could potentially take use of the opportunity to transfer the knowledge to patients to prevent new illnesses or even the illness of close family members living together. Second, the effect of health literacy on chronic disease prevention is not found in the rural sample, which implies the difference in accessing health facilities or health literacy promotion services among urban residents and rural residents might result in differences in health outcomes. Earlier diagnosis and education are more likely to help those with better socioeconomic background but appear to play a limited role among those who are poor and less educated. Third, family support could be a potential pathway of health literacy intervention. It is possible among patients with existing chronic conditions, an improvement on health literacy not only decreases the likelihood of developing an additional chronic condition for the individual, but also reduces the risk of a family member having a chronic condition.

## Conclusions

We usually observe lower chronic disease prevalence among people with higher level health literacy, this association, however, tells us little about the role health literacy plays in protecting individuals from chronic diseases. Our findings highlight the important role of health literacy in preventing comorbidities instead of preventing the first chronic disease. Moreover, family support can help to improve health literacy and result in beneficial effects on health. These findings extend our knowledge about health literacy and its effects on chronic disease prevention. Meanwhile, policymakers should improve equitable access of health literacy promotion services amongst all regions. Such promotions can be better designed to target patients with chronic diseases and their families to amplify the beneficial effects of health literacy.

## Supplementary information

**Additional file 1: Table S1.** Items in health literacy on chronic disease prevention. **Table S2.** Questions on chronic diseases in 2017 NHLS survey. **Table S3.** Additional information for the sample of respondents reporting having any chronic disease. **Table S4.** Summary statistics by level of health literacy on CDP – Urban and rural samples. **Tale S5.** OLS estimates on having any chronic disease - Urban sample. **Table S6.** OLS estimates on having any chronic disease - Rural sample. **Table S7.** OLS estimates on having any chronic disease by age group. **Table S8.** OLS estimates on having adequate health literacy on CDP: Full results. **Table S9.** Robustness tests on having comorbid chronic diseases. **Table S10.** Logit estimates on having any chronic disease (marginal effects). **Table S11.** Logit estimates on having adequate health literacy on CDP: Disease effects (marginal effects). **Table S12.** Robustness test using health literacy score on CDP for Table 3. **Table S13.** Robustness test using health literacy score on CDP for Table 4. **Table S14.** Robustness test using health literacy score on CDP for Table 5.

## Data Availability

The data used and/or analysed in the current study are not publicly available because restrictions apply to the availability of these data. Data are however available from the corresponding author on reasonable requests and with permission of Ningbo Municipal CDC.

## Abbreviations

NHLS: National Health Literacy Surveillance
WHO: World Health Organization
PPS: Probability Proportional to Size
PSU: Primary Sampling Unit
CDP: Chronic Disease Prevention
LPM: Linear Probability Model
SUR: Seemingly Unrelated Regression

## Footnotes

1 These figures come from 2012 European Health Literacy Survey, which was conducted in eight European countries: Austria, Bulgaria, Germany, Greece, Ireland, the Netherlands, Poland and Spain.

2 All neighborhood-committees within each street represent urban regions and all villages within each township represents rural regions.

3 In each household, all age-eligible (15–69 years) household members (i.e. who had been living there for more than 6 of the previous 12 months) was ordered by their gender and age. One member was then selected for the survey by means of a Kish grid [21].

4 An earlier paper published in a Chinese Journal reported the instruments used in the 2012 questionnaire has Cronbach’s alpha of 0.931 and split-half coefficient 0.808 [23].

5 Although not of interest to this paper per se, see [23] how comparability in questionnaires in 2008 and 2012 was achieved by pretesting three sets of questionnaires derived from a standardized question bank with the 2008 one using a group of participants that are representative of middle-income residents from both rural and urban areas.

6 The median income is 50,000 CNY ($ 7142).

7 Ningbo is next to Shanghai, a cosmopolitan city in China. In a study using 2017 NHLS data in Shanghai, the rate of health literacy on CDP is reported to be 24.2% [24].

8 This rate has largely kept constant from 2015 and 2016 surveys in Ningbo and is slightly higher than that in a neighbouring city by 2 percentage points, Qingdao, also a well-developed costal city in China [25]. Qingdao and Ningbo are often put together because of their similarities in many aspects, including population size, GDP, location, etc.

9 The information on the number of chronic diseases and the onset of the first chronic disease is reported in Table S3 in the Additional file 1. Among our respondents reporting having one or more chronic diseases, the majority had only one chronic condition, 15% reported having two types of diseases and 2% having more than two. About 36% of those with at least one type of chronic disease have the disease diagnosed in the past year and 30% had their first chronic disease diagnosed more than 4 years ago.

10 They are also more likely to self-report having ‘very good’ health. This variable is not reported but available upon request.

11 Another interpretation might be health literacy is important to explain the education difference in health outcomes.

12 We also tested whether there exists geographical clustering effect by including 112 dummies indicating the neighborhood-committee/village of the respondent. These dummies are jointly significantly at 1% significance level. Our main estimate of interest on health literacy on CDP remains significant at 10% significance level, and the effect gets slightly greater, to 0.030. These results are not reported, but available upon request.

13 The results in rural and urban samples do not differ significantly. Income appears a stronger predictor in the rural sample than in the urban sample and education appears to be a stronger predictor in the urban sample. In the urban sample, those who are illiterate and those who have an elementary education also differ significantly in having chronic diseases while in the rural sample, the two groups have similar likelihood of having chronic diseases.

14 Similar results are found for rural and urban sample.

15 In an alternative specification, we treat the number of chronic conditions as continuous, still we find insignificant results. These results are not reported, but available upon request.

16 We also considered alternative specification by additionally controlling for other disease conditions (adding 4 dummy variables indicating other chronic disease types) and find similar results.

17 We are also interested whether diabetes could result in similar interaction effect given its relatively high prevalence rate. The effect is only negative in predicting cerebrovascular disease but not statistically significant from zero.

18 We also tested whether the 112 dummies are jointly significant at 1% significance level and we reject the null hypothesis that all the coefficients are zero, suggesting there are clustering effect at neighbourhood-committee/village level.

19 0.651= 1-F(10,0,0.1)=1-(0.9)^10^ where F is binomial cumulative density function.

20 0.00149= F (10,5,0.1) where F is binomial cumulative density function. Strictly speaking, we should not count the interaction results in the last two columns as being ‘significant’. Despite this, the probability that three out of ten pairs showed up significant by chance would be 0.057, which is still smaller than the conventional 0.1 threshold.

21 Table 5 cannot be estimated using logit because we ran into perfect prediction case and the sample size reduces greatly. The interaction term is dropped and cannot be estimated.

22 Similar results are found for rural and urban sample in Tables S5 and S6 in the Additional file 1.
