# Supplementary material for: Health literacy and its effect on chronic disease prevention: evidence from China’s data
Source: BMC Public Health. 2020 May 14;20:690. doi: 10.1186/s12889-020-08804-4 (PMC7227325; doi:10.1186/s12889-020-08804-4)
Supplement: Supplementary file 1 — Additional file 1: Table S1. Items in health literacy on chronic disease prevention. Table S2. Questions on chronic diseases in 2017 NHLS survey. Table S3. Additional information for the sample of respondents reporting having any chronic disease. Table S4. Summary statistics by level of health literacy on CDP – Urban and rural samples. Tale S5. OLS estimates on having any chronic disease - Urban sample. Table S6. OLS estimates on having any chronic disease - Rural sample. Table S7. OLS estimates on having any chronic disease by age group. Table S8. OLS estimates on having adequate health literacy on CDP: Full results. Table S9. Robustness tests on having comorbid chronic diseases. Table S10. Logit estimates on having any chronic disease (marginal effects). Table S11. Logit estimates on having adequate health literacy on CDP: Disease effects (marginal effects). Table S12. Robustness test using health literacy score on CDP for Table 3. Table S13. Robustness test using health literacy score on CDP for Table 4. Table S14. Robustness test using health literacy score on CDP for Table 5. [file 12889_2020_8804_MOESM1_ESM.docx]

# Additional file

**Table S1** Items in health literacy on chronic disease prevention

| **Type of items** | **Item of Question** | **Dimension** | **% correct response**  **(n=8194)** |
| --- | --- | --- | --- |
| True/False | **A04** Nutrients in vegetables and fruits are similar; so vegetables can be replaced by fruits. | Knowledge and attitude | 74.3 |
| True/False | **A06** Adolescents can also have depression. | Knowledge and attitude | 84.5 |
| Single-answer | **B04** Which one is wrong regarding the self-monitored blood pressure?  (1) Self-monitored blood pressure can help doctors’ diagnosis;  (2) Patients with high blood pressure need to self-monitor their blood pressure regularly, and the records can be used as reference by doctors to design treatment plan and to assess the outcomes of the treatment;  (3) Patients with high blood pressure can be exempted from outpatient visits for regular follow-up check if their self-monitored blood pressure reads stable;  (4) I don’t know. | Behaviour and lifestyle | 54.7 |
| Single-answer | **B05** The following statements describe the danger of smoking, which one is incorrect:  (1) Tobacco dependence is an addictive chronic disease;  (2) Smoking can cause a number of chronic diseases;  (3) Low-tar cigarettes are less harmful than regular cigarettes;  (4) I don’t know. | Knowledge and attitude | 54.1 |
| Single-answer | **B06** Which one is not an early warning sign of cancer:   1. Find abnormal lumps in your body; 2. Find any blood in your stool; 3. Weight increase for no known reasons; 4. I don’t know. | Behaviour and lifestyle | 61.9 |
| Single-answer | **C09** Which of following describe the benefits of eating bean products such as bean-curd and soybean:  (1) Good for your health; (2) Of benefit to patients with heart diseases; (3) Source of good proteins; (4) It can be used as treatment for certain diseases; (5) I don’t know. | Knowledge and attitude | 37.7 |
| Single-answer | **C10** The benefits of regular exercise include：  (1) Maintain a healthy weight; (2) Reduce the risk of chronic diseases; (3) Reduce stress; (4) Help relaxation and sleep quality; (5) I don’t know. | Knowledge and attitude | 47.9 |
| Vignette Question | **D03** (A paragraph of introduction to body mass index is given before the question) Mr. Li is 45 years old and his BMI is 27.7 (kg/m^2^). To help Mr. Li control his weight, which of the following methods can be used? (More than one options apply):  (1) Do not eat rice; (2) Daily exercise for at least 30 min;  (3) Reduce fat intake; (4) Eat only vegetables and fruits;  (5) I don’t know. | Behaviour and lifestyle | 65.6 |
| Vignette question | **D04** (Same as above) Mr. Li is likely to have which one of the diseases below:  (1) High blood pressure; (2) Osteoporosis; (3) Stomach ulcer. | Behaviour and lifestyle | 73.4 |

**Table S2** Questions on chronic diseases in 2017 NHLS survey

|  | **Question** |
| --- | --- |
|  | **F09** Do you have any of the following chronic diseases? (choose all options that apply)  (1) I have no chronic disease 🡪 Skip to **F11**  (2) Hypertension (3) Heart diseases  (4) Cerebrovascular diseases (e.g. stroke, cerebral ischemic stroke and cerebral embolism)  (5) Diabetes (6) Malignant tumour (7) Other |
|  | **F10** As to your first chronic disease, for ______ year(s) have you been diagnosed with your first chronic disease (if less than one year, please fill ‘0.5’). |

| **Table S3** Additional information for the sample of respondents reporting having any chronic disease | | | | | | | |
| --- | --- | --- | --- | --- | --- | --- | --- |
| Sample | Has any chronic disease | | | | Rural | Urban | Rural vs Urban |
| Number of Observations | N=2120 | | | | n=1221 | n=899 |  |
| Variables | mean | s.d. | min/max | | mean | mean | p-value |
| *Number of chronic diseases* |  |  |  |  |  |  |  |
| 1:One disease | 0.837 | 0.370 | 0 | 1 | 0.835 | 0.839 | 0.838 |
| 2:Two diseases | 0.147 | 0.354 | 0 | 1 | 0.146 | 0.148 | 0.890 |
| 3:Three diseases | 0.015 | 0.122 | 0 | 1 | 0.018 | 0.011 | 0.198 |
| 4:Four diseases | 0.001 | 0.038 | 0 | 1 | 0.001 | 0.002 | 0.395 |
| *Diagnosis of first chronic disease* |  |  |  |  |  |  |  |
| 1:One year | 0.358 | 0.480 | 0 | 1 | 0.410 | 0.288 | 0.000 |
| 2:2-4 years | 0.349 | 0.477 | 0 | 1 | 0.342 | 0.358 | 0.427 |
| 3:5+ years | 0.293 | 0.455 | 0 | 1 | 0.249 | 0.354 | 0.000 |

Note: The p-value is calculated using either the t-test (if continuous) or the proportion test (if binary); a pre-test of equality of variance is also conducted.

| **Table S4** Summary statistics by level of health literacy on CDP – Urban and rural samples | | | | | | |
| --- | --- | --- | --- | --- | --- | --- |
| Sample | Urban | | | Rural | | |
|  | Health literacy | |  | Health literacy | |  |
| Subsamples | Adequate | Inadequate |  | Adequate | Inadequate |  |
| Number of observations | n=1144 | n=2858 |  | n=970 | n=3222 |  |
| Variables | mean | mean | p-value | mean | mean | p-value |
| **Key variables of interest** |  |  |  |  |  |  |
| Any chronic diseases (=1) | 0.195 | 0.239 | 0.002 | 0.261 | 0.303 | 0.012 |
| Hypertension (=1) | 0.142 | 0.170 | 0.031 | 0.189 | 0.223 | 0.024 |
| Heart problems (=1) | 0.016 | 0.019 | 0.543 | 0.028 | 0.019 | 0.078 |
| Cerebrovascular disease (=1) | 0.004 | 0.006 | 0.466 | 0.006 | 0.008 | 0.498 |
| Diabetes (=1) | 0.029 | 0.048 | 0.008 | 0.052 | 0.056 | 0.580 |
| Cancer (=1) | 0.007 | 0.006 | 0.705 | 0.006 | 0.009 | 0.355 |
| Other chronic diseases (=1) | 0.030 | 0.033 | 0.569 | 0.032 | 0.042 | 0.164 |
| Adequate health literacy on CDP^a^ (=1) | 1.000 | 0.000 | 0.000 | 1.000 | 0.000 | 0.000 |
| **Demographics** |  |  |  |  |  |  |
| Region (=1 urban) | 1.000 | 1.000 |  | 0.000 | 0.000 |  |
| Gender (=1 male) | 0.481 | 0.485 | 0.795 | 0.489 | 0.494 | 0.766 |
| Age in years | 42.865 | 47.763 | 0.000 | 48.032 | 52.377 | 0.000 |
| 1:Aged 15-44 | 0.560 | 0.395 | 0.000 | 0.336 | 0.218 | 0.000 |
| 2:Aged 45-59 | 0.309 | 0.362 | 0.001 | 0.422 | 0.432 | 0.555 |
| 3:Aged 60-69 | 0.131 | 0.242 | 0.000 | 0.242 | 0.349 | 0.000 |
| Household size | 2.910 | 2.715 | 0.000 | 2.962 | 2.852 | 0.025 |
| Annual income (1000 CNY) | 128.146 | 92.036 | 0.000 | 80.800 | 68.650 | 0.010 |
| **Education level** |  |  |  |  |  |  |
| 1:Illiterate | 0.019 | 0.046 | 0.000 | 0.094 | 0.143 | 0.000 |
| 2:Elementary | 0.093 | 0.214 | 0.000 | 0.276 | 0.391 | 0.000 |
| 3:Middle school | 0.207 | 0.324 | 0.000 | 0.315 | 0.307 | 0.628 |
| 4:High school | 0.244 | 0.213 | 0.036 | 0.162 | 0.103 | 0.000 |
| 5:College or above | 0.437 | 0.203 | 0.000 | 0.153 | 0.056 | 0.000 |
| **Occupation** |  |  |  |  |  |  |
| 1:Working in public sectors | 0.192 | 0.101 | 0.000 | 0.106 | 0.047 | 0.000 |
| 2:Farmers | 0.075 | 0.139 | 0.000 | 0.443 | 0.451 | 0.674 |
| 3:Manual labourers | 0.163 | 0.183 | 0.127 | 0.182 | 0.193 | 0.462 |
| 4:Working in private sectors | 0.318 | 0.229 | 0.000 | 0.105 | 0.091 | 0.174 |
| 5:Other | 0.252 | 0.348 | 0.000 | 0.163 | 0.218 | 0.000 |

Note: The p-value is calculated using either the t-test (if continuous) or the proportion test (if binary); a pre-test of equality of variance is also conducted. ^a^ CDP refers to chronic disease prevention.

| **Tale S5** OLS estimates on having any chronic disease - Urban sample | | | | | |
| --- | --- | --- | --- | --- | --- |
| Dep: Has any chronic disease | (1) | (2) | (3) | (4) | (5) |
| Sample | Urban | Urban | Urban | Urban | Urban |
| Adequate health literacy on CDP (=1) | -0.044*** | -0.035** | -0.018 | 0.024* | 0.024* |
|  | (0.015) | (0.015) | (0.015) | (0.014) | (0.014) |
| Gender (=1 male) |  | 0.000 |  |  | 0.004 |
|  |  | (0.013) |  |  | (0.012) |
| Annual income (log) |  | -0.018*** |  |  | -0.006* |
|  |  | (0.004) |  |  | (0.004) |
| Household size |  | -0.021*** |  |  | -0.003 |
|  |  | (0.004) |  |  | (0.004) |
| *Occupation (Base: 1:Public sectors)* |  |  |  |  |  |
| 2:Farmers |  |  | 0.259*** |  | 0.016 |
|  |  |  | (0.026) |  | (0.028) |
| 3:Manual labourers |  |  | 0.096*** |  | 0.023 |
|  |  |  | (0.024) |  | (0.024) |
| 4:Private sectors |  |  | 0.033 |  | 0.012 |
|  |  |  | (0.022) |  | (0.021) |
| 5:Other |  |  | 0.110*** |  | -0.008 |
|  |  |  | (0.022) |  | (0.022) |
| *Age group (Base: 1:Aged 15-44)* |  |  |  |  |  |
| 2:Aged 45-59 |  |  |  | 0.209*** | 0.210*** |
|  |  |  |  | (0.015) | (0.015) |
| 3:Aged 60-69 |  |  |  | 0.406*** | 0.406*** |
|  |  |  |  | (0.020) | (0.020) |
| *Education (Base: 1:Illiterate)* |  |  |  |  |  |
| 2:Elementary |  |  |  | -0.088*** | -0.087** |
|  |  |  |  | (0.034) | (0.034) |
| 3:Middle |  |  |  | -0.114*** | -0.110*** |
|  |  |  |  | (0.034) | (0.035) |
| 4:High school |  |  |  | -0.116*** | -0.111*** |
|  |  |  |  | (0.035) | (0.036) |
| 5:College or above |  |  |  | -0.136*** | -0.126*** |
|  |  |  |  | (0.036) | (0.038) |
| Observations | 4002 | 4002 | 4002 | 4002 | 4002 |
| R-squared | 0.002 | 0.014 | 0.033 | 0.168 | 0.170 |

Note: The dependent variable is a binary variable indicating whether a respondent has any chronic disease (=1 if has any chronic disease, 0 otherwise). Estimates on the constant are not reported. ***p<0.01, ** p<0.05, * p<0.1. Standard errors in parentheses.

| **Table S6** OLS estimates on having any chronic disease - Rural sample | | | | | |
| --- | --- | --- | --- | --- | --- |
| Dep: Has any chronic disease | (1) | (2) | (3) | (4) | (5) |
| Sample | Rural | Rural | Rural | Rural | Rural |
| Adequate health literacy on CDP (=1) | -0.042** | -0.035** | -0.032* | 0.013 | 0.010 |
|  | (0.017) | (0.016) | (0.017) | (0.016) | (0.016) |
| Gender (=1 male) |  | 0.003 |  |  | -0.005 |
|  |  | (0.014) |  |  | (0.013) |
| Annual income (log) |  | -0.031*** |  |  | -0.015*** |
|  |  | (0.005) |  |  | (0.005) |
| Household size |  | -0.033*** |  |  | -0.007 |
|  |  | (0.005) |  |  | (0.005) |
| *Occupation (Base: 1:Public sectors)* |  |  |  |  |  |
| 2:Farmers |  |  | 0.246*** |  | 0.027 |
|  |  |  | (0.030) |  | (0.033) |
| 3:Manual labourers |  |  | 0.122*** |  | 0.028 |
|  |  |  | (0.032) |  | (0.034) |
| 4:Private sectors |  |  | 0.036 |  | 0.010 |
|  |  |  | (0.036) |  | (0.035) |
| 5:Other |  |  | 0.137*** |  | -0.006 |
|  |  |  | (0.032) |  | (0.033) |
| *Age group (Base: 1:Aged 15-44)* |  |  |  |  |  |
| 2:Aged 45-59 |  |  |  | 0.190*** | 0.183*** |
|  |  |  |  | (0.019) | (0.020) |
| 3:Aged 60-69 |  |  |  | 0.395*** | 0.383*** |
|  |  |  |  | (0.022) | (0.023) |
| *Education (Base: 1:Illiterate)* |  |  |  |  |  |
| 2:Elementary |  |  |  | -0.007 | -0.001 |
|  |  |  |  | (0.021) | (0.022) |
| 3:Middle |  |  |  | -0.059** | -0.046* |
|  |  |  |  | (0.023) | (0.024) |
| 4:High school |  |  |  | -0.058** | -0.037 |
|  |  |  |  | (0.029) | (0.031) |
| 5:College or above |  |  |  | -0.074** | -0.046 |
|  |  |  |  | (0.035) | (0.038) |
| Observations | 4192 | 4192 | 4192 | 4192 | 4192 |
| R-squared | 0.001 | 0.026 | 0.034 | 0.133 | 0.137 |

Note: The dependent variable is a binary variable indicating whether a respondent has any chronic disease (=1 if has any chronic disease, 0 otherwise). Estimates on the constant are not reported. ***p<0.01, ** p<0.05, * p<0.1. Standard errors in parentheses.

| **Table S7** OLS estimates on having any chronic disease by age group | | | | |  |
| --- | --- | --- | --- | --- | --- |
| Dep: Has any chronic disease | (1) | (2) | (3) | (4) | |
| Sample | All | Aged 60-69 | Aged 45-59 | Aged 15-44 | |
| Adequate health literacy on CDP (=1) | 0.018* | 0.062** | 0.015 | -0.004 | |
|  | (0.011) | (0.028) | (0.019) | (0.010) | |
| Region (=1 urban) | 0.018* | 0.006 | 0.033* | 0.010 | |
|  | (0.010) | (0.024) | (0.018) | (0.010) | |
| Gender (=1 male) | -0.000 | -0.044* | 0.001 | 0.030*** | |
|  | (0.009) | (0.022) | (0.016) | (0.009) | |
| Annual income (log) | -0.010*** | -0.010 | -0.011** | -0.010*** | |
|  | (0.003) | (0.008) | (0.005) | (0.002) | |
| Household size | -0.005* | -0.009 | -0.010* | 0.002 | |
|  | (0.003) | (0.007) | (0.006) | (0.003) | |
| *Occupation (Base: 1:Public sectors)* |  |  |  |  | |
| 2:Farmers | 0.020 | -0.075 | 0.020 | 0.058*** | |
|  | (0.020) | (0.068) | (0.043) | (0.021) | |
| 3:Manual labourers | 0.023 | 0.001 | -0.005 | 0.032** | |
|  | (0.020) | (0.073) | (0.043) | (0.015) | |
| 4:Private sectors | 0.011 | -0.091 | 0.009 | 0.022* | |
|  | (0.018) | (0.075) | (0.043) | (0.013) | |
| 5:Other | -0.009 | -0.091 | -0.030 | 0.012 | |
|  | (0.019) | (0.068) | (0.042) | (0.014) | |
| *Age group (Base: 1:Aged 15-44)* |  |  |  |  | |
| 2:Aged 45-59 | 0.198*** |  |  |  | |
|  | (0.012) |  |  |  | |
| 3:Aged 60-69 | 0.397*** |  |  |  | |
|  | (0.015) |  |  |  | |
| *Education (Base: 1:Illiterate)* |  |  |  |  | |
| 2:Elementary | -0.024 | -0.025 | 0.002 | -0.107** | |
|  | (0.018) | (0.028) | (0.035) | (0.051) | |
| 3:Middle | -0.060*** | -0.047 | -0.050 | -0.147*** | |
|  | (0.019) | (0.036) | (0.034) | (0.048) | |
| 4:High school | -0.056*** | -0.039 | -0.045 | -0.145*** | |
|  | (0.022) | (0.057) | (0.039) | (0.049) | |
| 5:College or above | -0.071*** | -0.031 | -0.075 | -0.145*** | |
|  | (0.024) | (0.087) | (0.051) | (0.049) | |
| Observations | 8194 | 2203 | 3191 | 2800 | |
| R-squared | 0.155 | 0.010 | 0.008 | 0.021 | |

Note: The dependent variable is a binary variable indicating whether a respondent has any chronic disease (=1 if has any chronic disease, 0 otherwise). Estimates on the constant are not reported. ***p<0.01, ** p<0.05, * p<0.1. Standard errors in parentheses.

| **Table S8** OLS estimates on having adequate health literacy on CDP: Full results | | | | |
| --- | --- | --- | --- | --- |
| Dep: Adequate health literacy on CDP (=1) | (1) | (2) | (3) | (4) |
| Sample | Urban | Urban | Rural | Rural |
|  |  |  |  |  |
| Any chronic diseases (=1) | 0.031* |  | 0.010 |  |
|  | (0.018) |  | (0.015) |  |
| Duration first chronic disease: One year |  | 0.064** |  | 0.090*** |
|  |  | (0.029) |  | (0.021) |
| Duration first chronic disease: 2-4 years |  | 0.026 |  | -0.019 |
|  |  | (0.026) |  | (0.022) |
| Duration first chronic disease: 5+ years |  | 0.010 |  | -0.082*** |
|  |  | (0.027) |  | (0.026) |
| Gender (=1 male) | -0.008 | -0.009 | -0.012 | -0.012 |
|  | (0.014) | (0.014) | (0.013) | (0.013) |
| Annual income (log) | 0.005 | 0.005 | 0.001 | 0.002 |
|  | (0.004) | (0.004) | (0.004) | (0.004) |
| Household size | 0.008* | 0.008* | -0.003 | -0.003 |
|  | (0.005) | (0.005) | (0.004) | (0.004) |
| *Occupation (Base: 1:Public sectors)* |  |  |  |  |
| 2:Farmers | -0.050 | -0.054* | 0.005 | -0.000 |
|  | (0.032) | (0.032) | (0.032) | (0.032) |
| 3:Manual labourers | -0.045 | -0.044 | -0.045 | -0.046 |
|  | (0.027) | (0.027) | (0.033) | (0.033) |
| 4:Private sectors | -0.039 | -0.038 | -0.094*** | -0.095*** |
|  | (0.024) | (0.024) | (0.034) | (0.034) |
| 5:Other | -0.076*** | -0.077*** | -0.082** | -0.080** |
|  | (0.025) | (0.025) | (0.032) | (0.032) |
| *Age group (Base: 1:Aged 15-44)* |  |  |  |  |
| 2: Aged 45-59 | 0.002 | 0.002 | -0.012 | -0.013 |
|  | (0.018) | (0.018) | (0.019) | (0.019) |
| 3: Aged 60-69 | -0.022 | -0.018 | -0.048** | -0.046** |
|  | (0.024) | (0.024) | (0.023) | (0.023) |
| *Education (Base: 1:Illiterate)* |  |  |  |  |
| 2:Elementary | 0.004 | 0.009 | 0.017 | 0.021 |
|  | (0.039) | (0.039) | (0.021) | (0.021) |
| 3:Middle | 0.050 | 0.054 | 0.076*** | 0.074*** |
|  | (0.040) | (0.040) | (0.023) | (0.023) |
| 4:High school | 0.151*** | 0.156*** | 0.168*** | 0.163*** |
|  | (0.042) | (0.042) | (0.030) | (0.030) |
| 5:College or above | 0.285*** | 0.289*** | 0.295*** | 0.290*** |
|  | (0.043) | (0.044) | (0.037) | (0.037) |
| Observations | 4002 | 3994 | 4192 | 4185 |
| R-squared | 0.077 | 0.078 | 0.045 | 0.053 |

Note: Dependent variable is a binary variable indicating the level of health literacy (=1 if has adequate health literacy on CDP, 0 otherwise). Other covariates include gender, annual income, household members, occupation, age and education (and constant). Sample size differs in column (2) due to incomplete information provided by respondents on time since the first chronic disease was diagnosed. ***p<0.01, ** p<0.05, * p<0.1. Standard errors in parentheses.

| **Table S9** Robustness tests on having comorbid chronic diseases | | | | | |
| --- | --- | --- | --- | --- | --- |
| Dep: Has a specific chronic disease | Cerebro. | Cerebro. | Heart | Diabetes | Diabetes |
|  | (1) | (2) | (3) | (4) | (5) |
| Sample | Urban | Urban | Urban | Urban | Urban |
| **Panel A: Adding neighbourhood-committee/village FE** |  |  |  |  |  |
| Adequate health literacy on CDP (=1) | 0.002 | 0.001 | 0.007 | 0.003 | 0.003 |
|  | (0.003) | (0.003) | (0.005) | (0.008) | (0.008) |
| Heart problems (=1) | 0.064*** |  |  | 0.032 |  |
|  | (0.011) |  |  | (0.028) |  |
| Adequate health literacy on CDP $\times$ Heart problems (=1) | -0.076*** |  |  | -0.045 |  |
|  | (0.021) |  |  | (0.055) |  |
| Cancer |  | 0.050*** |  |  |  |
|  |  | (0.018) |  |  |  |
| Adequate health literacy on CDP $\times$ Cancer (=1) |  | -0.060* |  |  |  |
|  |  | (0.033) |  |  |  |
| Cerebrovascular disease (=1) |  |  | 0.184*** |  | 0.081* |
|  |  |  | (0.031) |  | (0.047) |
| Adequate health literacy on CDP $\times$ Cerebrovascular disease (=1) |  |  | -0.222*** |  | -0.164 |
|  |  |  | (0.066) |  | (0.100) |
| Neighbourhood-committee/Village FE | Yes | Yes | Yes | Yes | Yes |
|  |  |  |  |  |  |
| Observations | 4002 | 4002 | 4002 | 4002 | 4002 |
| R-squared | 0.029 | 0.022 | 0.042 | 0.058 | 0.059 |
| **Panel B: Weighted least squares** |  |  |  |  |  |
| Adequate health literacy on CDP (=1) | 0.001 | 0.001 | 0.005 | -0.001 | -0.001 |
|  | (0.003) | (0.003) | (0.005) | (0.007) | (0.007) |
| Heart problems (=1) | 0.064*** |  |  | 0.034 |  |
|  | (0.011) |  |  | (0.028) |  |
| Adequate health literacy on CDP $\times$ Heart problems (=1) | -0.072*** |  |  | -0.045 |  |
|  | (0.021) |  |  | (0.055) |  |
| Cancer |  | 0.049*** |  |  |  |
|  |  | (0.018) |  |  |  |
| Adequate health literacy on CDP $\times$ Cancer (=1) |  | -0.059* |  |  |  |
|  |  | (0.032) |  |  |  |
| Cerebrovascular disease (=1) |  |  | 0.187*** |  | 0.092* |
|  |  |  | (0.031) |  | (0.047) |
| Adequate health literacy on CDP $\times$ Cerebrovascular disease (=1) |  |  | -0.234*** |  | -0.164 |
|  |  |  | (0.066) |  | (0.100) |
| Observations | 4002 | 4002 | 4002 | 4002 | 4002 |
| R-squared | 0.018 | 0.011 | 0.032 | 0.037 | 0.037 |

Note: The dependent variable is a binary indicating whether the respondent has a specific chronic disease (e.g. =1 if has cerebrovascular disease, 0 otherwise in in column 1). Other covariates in each column include gender, annual income, household members, occupation, age and education (and constant). ***p<0.01, ** p<0.05, * p<0.1. Standard errors in parentheses.

| **Table S10** Logit estimates on having any chronic disease (marginal effects) | | | | | |
| --- | --- | --- | --- | --- | --- |
| Dep: Has any chronic disease | (1) | (2) | (3) | (4) | (5) |
| Sample | All | All | All | All | All |
| Adequate health literacy on CDP (=1) | -0.048*** | -0.033*** | -0.026** | 0.020* | 0.019* |
|  | (0.011) | (0.011) | (0.011) | (0.011) | (0.011) |
| Region (=1 urban) |  | -0.062*** |  |  | 0.018* |
|  |  | (0.010) |  |  | (0.010) |
| Gender (=1 male) |  | 0.001 |  |  | 0.000 |
|  |  | (0.010) |  |  | (0.009) |
| Annual income (log) |  | -0.019*** |  |  | -0.011*** |
|  |  | (0.003) |  |  | (0.003) |
| Household size |  | -0.040*** |  |  | -0.005 |
|  |  | (0.004) |  |  | (0.003) |
| *Occupation (Base: 1:Public sectors)* |  |  |  |  |  |
| 2:Farmers |  |  | 0.246*** |  | 0.018 |
|  |  |  | (0.016) |  | (0.023) |
| 3:Manual labourers |  |  | 0.110*** |  | 0.025 |
|  |  |  | (0.017) |  | (0.023) |
| 4:Private sectors |  |  | 0.035** |  | 0.014 |
|  |  |  | (0.016) |  | (0.023) |
| 5:Other |  |  | 0.121*** |  | -0.007 |
|  |  |  | (0.016) |  | (0.022) |
| *Age group (Base: 1:Aged 15-44)* |  |  |  |  |  |
| 2:Aged 45-59 |  |  |  | 0.208*** | 0.208*** |
|  |  |  |  | (0.010) | (0.010) |
| 3:Aged 60-69 |  |  |  | 0.405*** | 0.400*** |
|  |  |  |  | (0.015) | (0.015) |
| *Education (Base: 1:Illiterate)* |  |  |  | -0.018 | -0.017 |
| 2:Elementary |  |  |  |  |  |
|  |  |  |  | (0.016) | (0.016) |
| 3:Middle |  |  |  | -0.051*** | -0.047*** |
|  |  |  |  | (0.017) | (0.018) |
| 4:High school |  |  |  | -0.049** | -0.044** |
|  |  |  |  | (0.020) | (0.021) |
| 5:College or above |  |  |  | -0.081*** | -0.073*** |
|  |  |  |  | (0.023) | (0.026) |
| Observations | 8194 | 8194 | 8194 | 8194 | 8194 |
| Pseudo R-squared | 0.002 | 0.024 | 0.034 | 0.145 | 0.148 |

Note: The dependent variable is a binary variable indicating whether a respondent has any chronic disease (=1 if has any chronic disease, 0 otherwise). Estimates on the constant are not reported. ***p<0.01, ** p<0.05, * p<0.1. Standard errors in parentheses.

| **Table S11** Logit estimates on having adequate health literacy on CDP: Disease effects (marginal effects) | | | | |
| --- | --- | --- | --- | --- |
| Dep: Adequate health literacy on CDP (=1) | (1) | (2) | (3) | (4) |
| **Panel A: Urban sample** |  |  |  |  |
| Any chronic diseases (=1) | 0.035* |  |  |  |
|  | (0.019) |  |  |  |
| Duration first chronic disease: One year |  | 0.070** |  |  |
|  |  | (0.032) |  |  |
| Duration first chronic disease: 2-4 years |  | 0.028 |  |  |
|  |  | (0.028) |  |  |
| Duration first chronic disease: 5+ years |  | 0.011 |  |  |
|  |  | (0.030) |  |  |
| One disease |  |  | -0.032 |  |
|  |  |  | (0.020) |  |
| Two+ diseases |  |  | 0.020 |  |
|  |  |  | (0.044) |  |
| Hypertension (=1) |  |  |  | 0.048** |
|  |  |  |  | (0.023) |
| Observations | 4002 | 3994 | 4002 | 4002 |
| R-squared | 0.065 | 0.065 | 0.065 | 0.065 |
| **Panel B: Rural Sample** |  |  |  |  |
| Any chronic diseases (=1) | 0.011 |  |  |  |
|  | (0.016) |  |  |  |
| Duration first chronic disease: One year |  | 0.094*** |  |  |
|  |  | (0.023) |  |  |
| Duration first chronic disease: 2-4 years |  | -0.021 |  |  |
|  |  | (0.022) |  |  |
| Duration first chronic disease: 5+ years |  | -0.099*** |  |  |
|  |  | (0.023) |  |  |
| One disease |  |  | -0.004 |  |
|  |  |  | (0.016) |  |
| Two+ diseases |  |  | 0.044 |  |
|  |  |  | (0.036) |  |
| Heart problems (=1) |  |  |  | 0.127** |
|  |  |  |  | (0.052) |
| Observations | 4192 | 4185 | 4192 | 4192 |
| Pseudo R-squared | 0.040 | 0.049 | 0.040 | 0.042 |

Note: Dependent variable is a binary variable indicating the level of health literacy (=1 if has adequate health literacy on CDP, 0 otherwise). Other covariates include gender, annual income, household members, occupation, age and education (and constant). Full list of disease types in column (4) include hypertension, heart problems, cerebrovascular disease, diabetes and cancer and other diseases. Sample size differs in column (2) due to incomplete information provided by respondents on time since the first chronic disease was diagnosed. ***p<0.01, ** p<0.05, * p<0.1. Standard errors in parentheses.

| **Table S12** Robustness test using health literacy score on CDP for Table 3 | | | | | |
| --- | --- | --- | --- | --- | --- |
| Dep: Any chronic disease (=1) | (1) | (2) | (3) | (4) | (5) |
| Sample | All | All | All | All | All |
| Health literacy score on CDP (range 0-12) | -0.012*** | -0.009*** | -0.008*** | 0.001 | 0.001 |
|  | (0.002) | (0.002) | (0.002) | (0.002) | (0.002) |
| Region (=1 urban) |  | -0.054*** |  |  | 0.017* |
|  |  | (0.010) |  |  | (0.010) |
| Gender (=1 male) |  | 0.002 |  |  | -0.000 |
|  |  | (0.010) |  |  | (0.009) |
| Annual income (log) |  | -0.023*** |  |  | -0.010*** |
|  |  | (0.003) |  |  | (0.003) |
| Household size |  | -0.026*** |  |  | -0.005* |
|  |  | (0.003) |  |  | (0.003) |
| *Occupation (Base: 1:Public sectors)* |  |  |  |  |  |
| 2:Farmers |  |  | 0.234*** |  | 0.020 |
|  |  |  | (0.018) |  | (0.020) |
| 3:Manual labourers |  |  | 0.100*** |  | 0.023 |
|  |  |  | (0.019) |  | (0.020) |
| 4:Private sectors |  |  | 0.031 |  | 0.011 |
|  |  |  | (0.019) |  | (0.018) |
| 5:Other |  |  | 0.111*** |  | -0.010 |
|  |  |  | (0.018) |  | (0.019) |
| *Age group (Base: 1:Aged 15-44)* |  |  |  |  |  |
| 2:Aged 45-59 |  |  |  | 0.200*** | 0.198*** |
|  |  |  |  | (0.012) | (0.012) |
| 3:Aged 60-69 |  |  |  | 0.403*** | 0.397*** |
|  |  |  |  | (0.015) | (0.015) |
| *Education (Base: 1:Illiterate)* |  |  |  |  |  |
| 2:Elementary |  |  |  | -0.026 | -0.024 |
|  |  |  |  | (0.018) | (0.018) |
| 3:Middle |  |  |  | -0.065*** | -0.060*** |
|  |  |  |  | (0.018) | (0.019) |
| 4:High school |  |  |  | -0.062*** | -0.055** |
|  |  |  |  | (0.021) | (0.022) |
| 5:College or above |  |  |  | -0.079*** | -0.068*** |
|  |  |  |  | (0.022) | (0.025) |
| Observations | 8194 | 8194 | 8194 | 8194 | 8194 |
| Pseudo R-squared | 0.008 | 0.028 | 0.041 | 0.153 | 0.155 |

Note: The dependent variable is a binary variable indicating whether a respondent has any chronic disease (=1 if has any chronic disease, 0 otherwise). Estimates on the constant are not reported. ***p<0.01, ** p<0.05, * p<0.1. Standard errors in parentheses.

| **Table S13** Robustness test using health literacy score on CDP for Table 4 | | | | |
| --- | --- | --- | --- | --- |
| Dep: Health literacy score on CDP (range 0-12) | (1) | (2) | (3) | (4) |
| **Panel A: Urban sample** |  |  |  |  |
| Any chronic diseases (=1) | 0.109 |  |  |  |
|  | (0.111) |  |  |  |
| Duration first chronic disease: One year |  | 0.055 |  |  |
|  |  | (0.179) |  |  |
| Duration first chronic disease: 2-4 years |  | 0.116 |  |  |
|  |  | (0.162) |  |  |
| Duration first chronic disease: 5+ years |  | 0.190 |  |  |
|  |  | (0.169) |  |  |
| One disease |  |  | -0.133 |  |
|  |  |  | (0.117) |  |
| Two+ diseases |  |  | -0.165 |  |
|  |  |  | (0.243) |  |
| Hypertension (=1) |  |  |  | 0.340*** |
|  |  |  |  | (0.127) |
| Observations | 4002 | 3994 | 4002 | 4002 |
| R-squared | 0.126 | 0.127 | 0.126 | 0.130 |
| **Panel B: Rural Sample** |  |  |  |  |
| Any chronic diseases (=1) | -0.013 |  |  |  |
|  | (0.112) |  |  |  |
| Duration first chronic disease: One year |  | 0.770*** |  |  |
|  |  | (0.153) |  |  |
| Duration first chronic disease: 2-4 years |  | -0.273* |  |  |
|  |  | (0.164) |  |  |
| Duration first chronic disease: 5+ years |  | -0.986*** |  |  |
|  |  | (0.190) |  |  |
| One disease |  |  | 0.071 |  |
|  |  |  | (0.118) |  |
| Two+ diseases |  |  | 0.385 |  |
|  |  |  | (0.238) |  |
| Heart problems (=1) |  |  |  | 1.307*** |
|  |  |  |  | (0.337) |
| Observations | 4192 | 4185 | 4192 | 4192 |
| Pseudo R-squared | 0.093 | 0.107 | 0.094 | 0.097 |

Note: Dependent variable is a binary variable indicating the level of health literacy (=1 if has adequate health literacy on CDP, 0 otherwise). Other covariates include gender, annual income, household members, occupation, age and education (and constant). Full list of disease types in column (4) include hypertension, heart problems, cerebrovascular disease, diabetes and cancer and other diseases. Sample size differs in column (2) due to incomplete information provided by respondents on time since the first chronic disease was diagnosed. ***p<0.01, ** p<0.05, * p<0.1. Standard errors in parentheses.

| **Table S14** Robustness test using health literacy score on CDP for Table 5 | | | | | |
| --- | --- | --- | --- | --- | --- |
| Dep: Has a specific chronic disease | Cerebro. | Cerebro. | Heart | Diabetes | Diabetes |
|  | (1) | (2) | (3) | (4) | (5) |
| Sample | Urban | Urban | Urban | Urban | Urban |
| Health literacy score (range 0-12) | 0.000 | 0.000 | -0.000 | 0.000 | -0.000 |
|  | (0.000) | (0.000) | (0.001) | (0.001) | (0.001) |
| Heart problems (=1) | 0.078*** |  |  | 0.079 |  |
|  | (0.020) |  |  | (0.054) |  |
| Health literacy score$\times$ Heart problems (=1) | -0.005* |  |  | -0.009 |  |
|  | (0.003) |  |  | (0.007) |  |
| Cancer |  | 0.164*** |  |  |  |
|  |  | (0.049) |  |  |  |
| Health literacy score$\times$ Cancer (=1) |  | -0.017*** |  |  |  |
|  |  | (0.006) |  |  |  |
| Cerebrovascular disease (=1) |  |  | 0.377*** |  | 0.298** |
|  |  |  | (0.078) |  | (0.119) |
| Health literacy score $\times$ Cerebrovascular disease (=1) |  |  | -0.035*** |  | -0.036** |
|  |  |  | (0.011) |  | (0.016) |
| Observations | 4002 | 4002 | 4002 | 4002 | 4002 |
| R-squared | 0.016 | 0.012 | 0.031 | 0.037 | 0.038 |

Note: The dependent variable is a binary indicating whether the respondent has a specific chronic disease (e.g. =1 if has cerebrovascular disease, 0 otherwise in in column 1). Other covariates in each column include gender, annual income, household members, occupation, age and education (and constant). ***p<0.01, ** p<0.05, * p<0.1. Standard errors in parentheses.
